# Supplementary material for: The effects of a comparatively higher dose of 1000 mg/kg/d of oral L- or D-arginine on the L-arginine metabolic pathways in male Sprague-Dawley rats
Source: PLoS One. 2023 Aug 1;18(8):e0289476. doi: 10.1371/journal.pone.0289476 (PMC10393177; doi:10.1371/journal.pone.0289476)
Supplement: S1 Raw images — (PDF) [file pone.0289476.s001.pdf]

The effects of a comparatively higher dose of 1000 mg/kg/d of oral L- or D-arginine on the L-arginine metabolic pathways in male Sprague-Dawley rats.

Dain (Raina) Kim, Sarah Martin, Kaushik Desai

Western blots raw images

Images were captured with ChemiDoc Imaging System (G:BOX Chemi XX6, Syngene, Frederick, MD, USA). The protein bands on the image were manually quantified using GeneTools software (Syngene, Frederick, MD, USA). For loading control, the Invitrogen No-Stain Protein Labelling Reagent (Cat # A44449, Fisher Scientific) was used to perform total protein normalization.

Cationic transporter 1 (CAT-1) Figure 2A

Upper small intestine

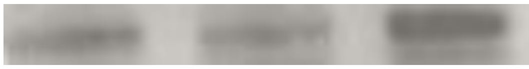

67kD

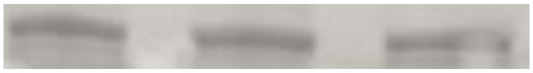

Total protein

Liver

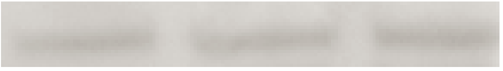

67 kD

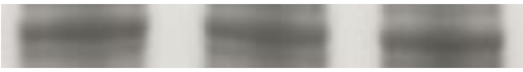

Total protein

Aorta

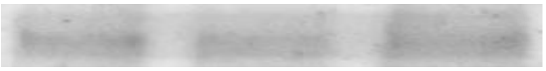

67 kD

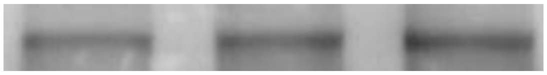

Total protein

Arginase I and II Figure 3A

Liver (Argn I)

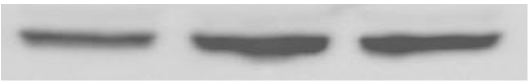

37 kD

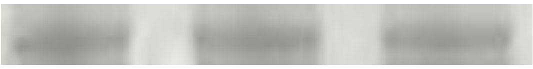

Total protein

Upper small intestine (Argn II)

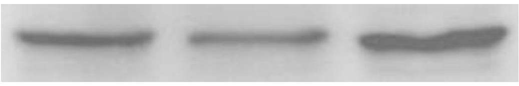

39 kD

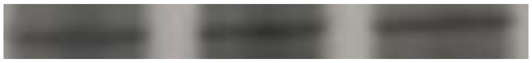

Total protein

Kidney (Argn II)

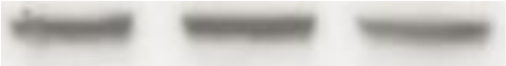

39 kD

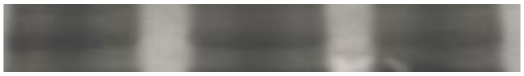

Total protein

Endothelial nitric oxide synthase (eNOS) Figure 4A

Aorta

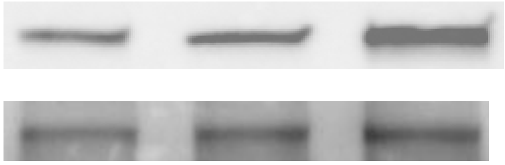

133 kD

Total protein

Brain

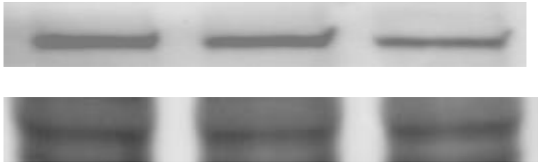

133 kD

Total protein

Kidney

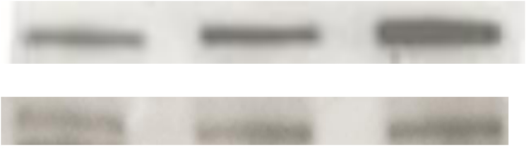

133 kD

Total protein

Arginine:glycine amidinotransferase (AGAT) Figure 6A

Liver

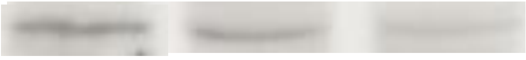

44 kD

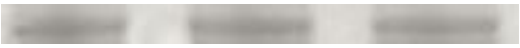

Total protein

Kidney

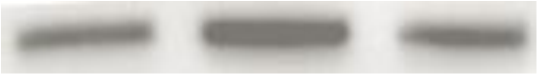

44 kD

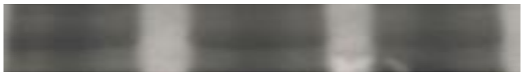

Total protein

Upper small intestine

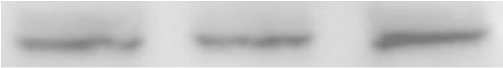

44 kD

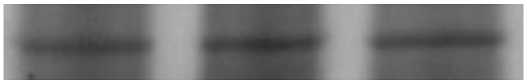

Total protein

Brain

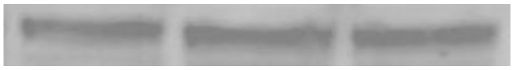

44 kD

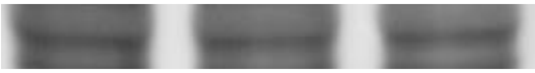

Total protein

Arginine decarboxylase (ADC) **Figure 7A**

Liver

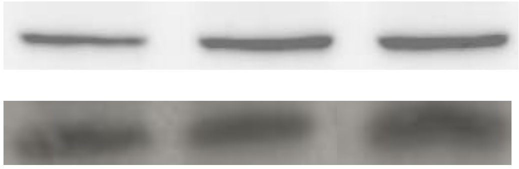

50 kD

Total protein

Kidney

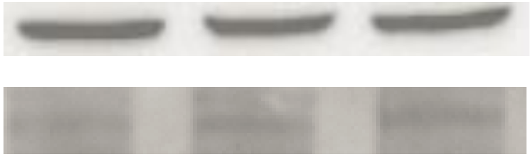

50 kD

Total protein

Upper small intestine

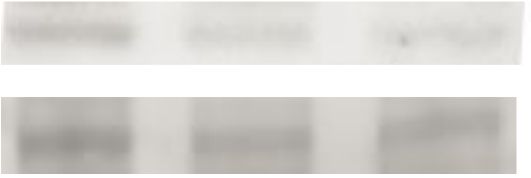

50 kD

Total protein

Brain

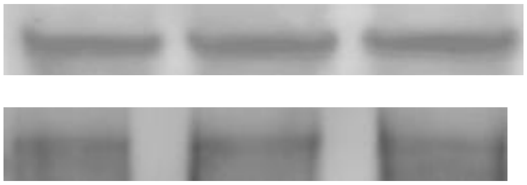

50 kD

Total protein

Agmatinase

Liver

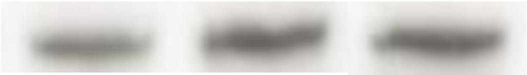

38 kD

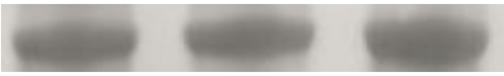

Total protein

Kidney

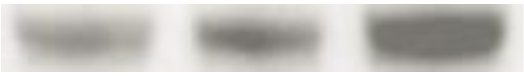

38 kD

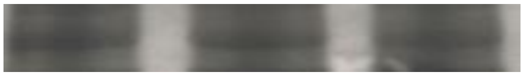

Total protein

Upper small intestine

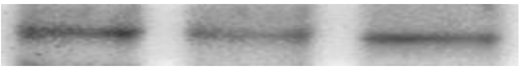

38 kD

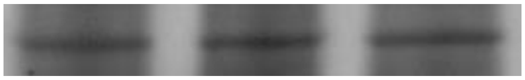

Total protein

Brain

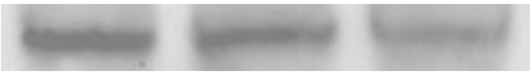

38 kD

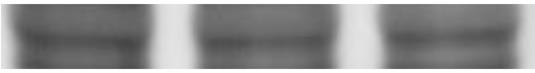

Total protein
